# Association between life’s essential 8 and male biochemical androgen deficiency: evidence from NHANES 2013–2016

**DOI:** 10.3389/fendo.2024.1369684

**Published:** 2024-06-24

**Authors:** Weisheng Huang, Mutong Chen, Haiyu Zhang, Zhongfu Zhang, Cong Yin, Meiyang Huang, Bentao Shi

**Affiliations:** ^1^ Department of Urology, the First Affiliated Hospital of Shenzhen University/Shenzhen Second People’s Hospital, Shenzhen, China; ^2^ Department of Clinical Medicine, Shantou University Medical College, Shantou, China

**Keywords:** life’s essential 8, biochemical androgen deficiency, endocrine function, reproduction, NHANES

## Abstract

**Purpose:**

To evaluate the association of Life’s Essential 8 (LE8) and its subscales with male biochemical androgen deficiency (MBAD) and total testosterone based on the data from the national health and nutrition examination survey (NHANES) database.

**Methods:**

Data of males aged 20 years or older from NHANES of 2013–2016 were extracted. LE8 score was calculated based on American Heart Association definitions. Total testosterone (TT) values were measured in NHANES using precise isotope dilution liquid chromatography. MBAD was defined as serum TT of <300 ng/dL. Univariate and multivariable analyses were conducted. Propensity score matching (PSM) and weighted regression after matching were added as sensitivity analyses. The generalized additive model, smooth curve fitting, and the recursive algorithm were used to determine the potential inflection points. Piecewise regression models with log-likelihood ratio test were used to quantify nonlinear effects.

**Results:**

A total of 3094 participants who were males and aged 20 years or above were included. Out of them, 805 males were diagnosed with MBAD. After adjusting the confounders in the multivariable model, LE8 was independently associated with MBAD (OR 0.96, P < 0.001) and TT (β 2.7, P < 0.001). The association remained robust even after PSM. The non-linear relationship of LE8 behaviors score with MBAD and TT was revealed.

**Conclusion:**

LE8 was an independent protective factor of MBAD and a feasible approach to promote male endocrine sexual function.

## 1 Introduction

Testosterone is the primary androgen in males. In males, 95% of testosterone is produced in Leyden cells of the testis under the regulation of the hypothalamic-pituitary-testis axis (1). Testosterone is necessary for spermatogenesis, stimulation of sexual desire, and normal sexual function in adults. Additionally, testosterone is involved in body composition, bone and muscle formation, erythropoiesis, and iron metabolism. By 2025, it is projected that the United States will witness a prevalence of symptomatic testosterone deficiency (TD) in approximately 6.5 million males (2). The American Urological Association guideline defined TD as testosterone levels lower than 300 ng/dL with corresponding symptoms or signs (3). It manifests as impaired development of muscle and body hair, gynecomastia, decreased stature, erectile dysfunction, and sexual difficulties (4).

In 2010, the American Heart Association (AHA) introduced the concept of Life’s Simple 7 (LS7) as a set of seven key indicators of cardiovascular health (CVH) that can be targeted for the improvement of cardiovascular well-being (5). Recently, the AHA has updated LS7, incorporating sleep health as an essential component, resulting in the formulation of Life’s Essential 8 (LE8) (6). LE8 comprises four behaviors: diet, physical activity, nicotine exposure, and sleep health, along with four factors: body mass index (BMI), blood lipids, blood glucose, and blood pressure.

The AHA’s LE8 construct is a useful tool for assessing and improving CVH. By following the health behaviors and health factors outlined in LE8, individuals can lower their risk for heart disease, stroke, and other major health problems. Higher LE8 scores have been associated with a lower risk of cardiovascular disease (CVD) and all-cause mortality among aging men (7). An analysis found that people whose lifestyles better reflect CVH can reap gains in life expectancy free from major chronic disease (8). Other researchers have reported that a high LE8 score is significantly associated with a lower risk of chronic kidney disease and nonalcoholic fatty liver disease (9, 10).

LE8 is a comprehensive index and numerous studies have demonstrated its association with various health conditions. Testosterone has been linked to CVH and its risk factors in multiple research (1, 11). Given the established connection, this study aims to investigate the association of LE8 with testosterone, an area that remains unexplored.

## 2 Materials and methods

### 2.1 Cohorts and participants

NHANES is an ongoing series of surveys conducted by the National Center for Health Statistics at the U.S. Centers for Disease Control and Prevention. Its purpose is to assess the health and nutritional status of the U.S. population through interviews and physical examinations (http://www.cdc.gov/nchs/nhanes.htm). NHANES follows a complex, multistage probability sampling design in a 2-year cycle to ensure nationally representative data for the civilian noninstitutionalized U.S. population. More detailed information about NHANES methods and protocols can be found on their website. The NCHS Research Ethics Review Board approved the NHANES procedures and protocols, with written informed consent obtained from all participants. The reporting guidelines of Strengthening the Reporting of Observational Studies in Epidemiology were followed during this study.

**Figure 1:**
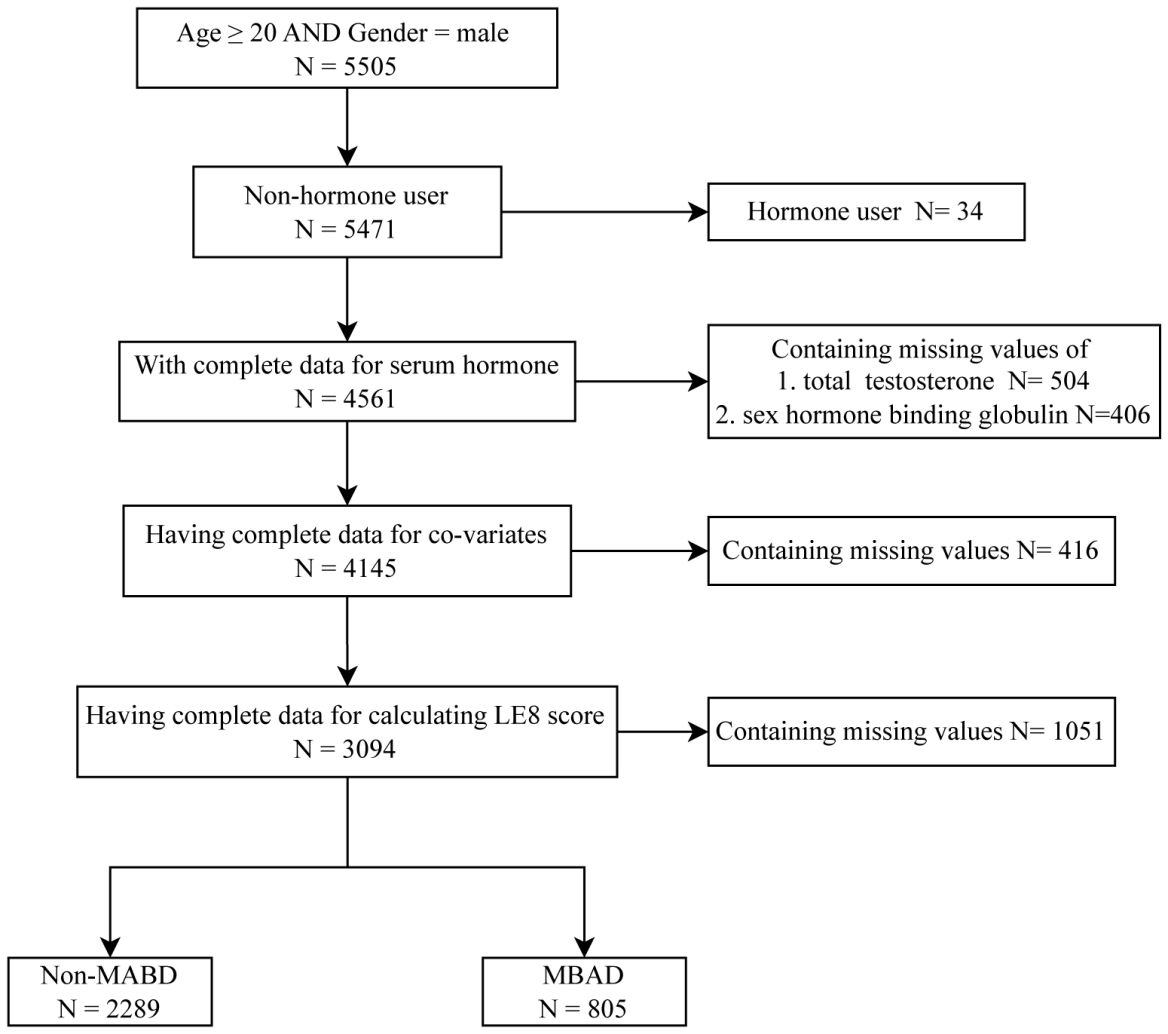
Flow diagram of the screening and enrollment of study participants.

Data were limited to the continuous data cycles of 2013–2014 and 2015–2016. The cohort consisted of men aged 20 years who completed a comprehensive 24-hour dietary history and underwent sex hormone testing. Participants with incomplete data regarding sex hormones and dietary recall assessments were excluded from the analysis. After further exclusion of men taking medication related to sex hormones, such as testosterone, progesterone, estrogen, or other similar substances mentioned in the NHANES questionnaire, a total of 3,094 participants were included in the study (**Figure 1**).

### 2.2 Measurement of LE8

The LE8 score comprises 4 health behaviors (diet, physical activity, nicotine exposure, and sleep duration) and 4 health factors (BMI, non-high-density lipoprotein (non-HDL) cholesterol, blood glucose, and blood pressure). Dietary indicators were assessed employing the Healthy Eating Index (HEI) 2015 measured by the subjects’ 24-hour dietary review (12). Relevant information such as physical activity, nicotine exposure, sleep patterns, diabetes history, and medication usage were obtained via a self-report survey. Measurements of height, weight, and blood pressure were conducted in the physical examination. The BMI was calculated by dividing the weight (kilograms) by the height (meters) squared. Non–HDL cholesterol, plasma glucose, and hemoglobin A1c were measured from collected blood samples. The algorithm for determining the LE8 score in the NHANES dataset has been previously documented (6, 13). Each of the 8 CVH indicators was assigned a score between 0 and 100, and the overall LE8 score was calculated as an average of these indicators. Meanwhile, individuals with high CVH were categorized as having a LE8 score ranging from 80 to 100. Moderate CVH was defined as a LE8 score between 50 and 79, while low CVH referred to a LE8 score of 0 to 49 (6). The same cut-off points were applied in our research for classifying the subscales.

### 2.3 Measurement and definition of outcomes

The primary outcomes were total testosterone (TT), free testosterone (FT), and male biochemical androgen deficiency (MBAD). TT values were measured in NHANES using precise isotope dilution liquid chromatography and tandem mass spectrometry at a single time point in the morning, afternoon, or evening. FT was calculated based on the Vermeulen Equation using the online tool Free & Bioavailable Testosterone calculator (www.issam.ch/freetesto.html) (14). MBAD was defined as serum TT of <300 ng/dL in this study according to the American Urological Association guidelines (3). Given that NHANES does not comprehensively include the symptoms and physical signs necessary for evaluating TD, and questionnaires are not recommended for reporting clinical symptoms (3, 15), this study establishes the testosterone threshold as the sole inclusion criterion for MBAD.

### 2.4 Study covariates

Potential variables confounding the association between LE8 and MBAD were included in multivariable models. The covariates included age (16–18), ethnicity (19), education level, marital status, poverty ratio, time of sample collection (venipuncture time), and self-report cardiovascular disease (CVD). Education level was classified as high school or less, some college and college graduate or above. marital status was classified as married/living with a partner, never married, and widowed/divorced/separated. The poverty ratio was categorized as ≤1.3, 1.4–3.5, and >3.5. Considering that testosterone may have circadian variation (20), the venipuncture time was categorized as morning, afternoon, or evening. CVD was defined as a self-reported history of one of the following conditions: coronary heart disease, myocardial infarction, congestive heart failure, and stroke.

### 2.5 Statistical analysis

Weighted mean, weighted proportions, and corresponding 95% confidence interval (CI) were used to describe the characteristics of the participants. Continuous data were compared by survey t-test, and categorical data were compared by Rao-Scott chi-square test. Odds ratio (OR), β, and corresponding 95% CI were calculated. Weighted histograms were employed in this study to assess whether the data conform to a normal distribution (**Supplementary Figure S1**). Ultimately, only FT was observed to exhibit pronounced non-normality, prompting us to perform a logarithmic transformation (log2) and include it in our sensitivity analysis (**Supplementary Tables S7**, **S8**). Given the lack of knowledge regarding the overall population distribution of LE8 scores, we refrained from transforming them. During regression analysis, we utilized both the original LE8 scores and a segmented version suitable for left-skewed distributions. Weighted logistic regression model and weighted linear regression were employed to calculate the independent associations of LE8 as well as its subscales with TT, FT, and MBAD. Three models were constructed with model 1 being a crude model without adjustment for covariate, model 2 adjusted for venipuncture time, age, and ethnicity, and model 3 further adjusted for marital income level, education, and self-reported CVD history of one of the following conditions: coronary heart disease, heart attack, congestive heart failure, or stroke. LE8 score was categorized according to the scoring intervals mentioned previously. The generalized additive model, smooth curve fitting, and the recursive algorithm were used to determine the potential inflection points. Piecewise regression models with log-likelihood ratio test were used to quantify nonlinear effects. A 1:1 propensity score matching (PSM) and weighted regression after matching were added as sensitivity analyses. Furthermore, given the potential impact of mental distress on hormone levels (21, 22), we excluded individuals taking psychiatric drugs and further controlled for Patient Health Questionnaire (PHQ-9) score which measures the severity of depression in subsequent sensitivity analyses. The software R 4.3.1 and Empower^®^ (www.empowerstats.com) were utilized for conducting all statistical analyses. Two-tailed p values <0.05 were considered statistically significant.

## 3 Results

### 3.1 Baseline characteristics

In the NHANES Continuous Surveys from 2013–2016, 3094 individuals who were males and aged 20 years or above were considered for inclusion. Baseline characteristics of them were presented in **Table 1**, categorized according to their MBAD status. Before PSM, 805 males were diagnosed with MBAD. Compared to those without MBAD, participants with MBAD tended to be older and coupled and have a CVD history. The LE8 score (70.50, 95%CI 69.45–71.55) and LE8 health factors score (72.42, 95%CI 71.09–73.75) were higher in participants without MBAD, while there was no significant difference in the LE8 health behaviors score between the two groups. A total of 1610 participants were enrolled after PSM, including 805 participants with and without MBAD. Except for the study variables, other demographic characteristics were controlled for almost the same level. The homogeneity was almost achieved in these two groups (**Table 1**; **Supplementary Figure S2**).

**Table 1:** Baseline characteristics of the study population with and without MBAD.

	Before PSM			After PSM		
	Non MBAD (n=2289)	MBAD (n=805)	*P*	Non MBAD (n=805)	MBAD (n=805)	*P*
Age, years	46.09 (44.97,47.22)	49.28 (47.49,51.08)	**0.01**	50.17 (48.82,51.52)	49.28 (47.49,51.08)	0.47
Age strata			**0.01**			0.94
[20,40)	41.72 (38.85,44.60)	31.75 (26.06,37.44)		29.72 (24.79,34.64)	31.75 (26.06,37.44)	
[40,60)	32.71 (29.80,35.63)	40.57 (34.68,46.45)		39.42 (33.91,44.92)	40.57 (34.68,46.45)	
[60,)	25.56 (22.48,28.64)	27.68 (22.51,32.86)		30.86 (26.62,35.10)	27.68 (22.51,32.86)	
Time of venipuncture			**< 0.0001**			0.96
Afternoon	34.76 (31.81,37.71)	43.61 (39.26,47.97)		48.53 (43.18,53.87)	43.61 (39.26,47.97)	
Evening	18.00 (14.96,21.03)	28.16 (23.62,32.70)		22.57 (17.50,27.64)	28.16 (23.62,32.70)	
Morning	47.25 (45.18,49.32)	28.23 (22.74,33.71)		28.90 (25.48,32.32)	28.23 (22.74,33.71)	
Ethnicity			0.23			0.94
Mexican American	9.24 (6.62,11.87)	9.45 (6.40,12.51)		10.13 (7.10,13.16)	9.45 (6.40,12.51)	
Non-Hispanic Black	10.45 (7.94,12.97)	7.22 (4.60, 9.83)		8.86 (6.29,11.44)	7.22 (4.60, 9.83)	
Non-Hispanic White	65.49 (60.59,70.40)	69.43 (63.90,74.97)		68.30 (62.81,73.80)	69.43 (63.90,74.97)	
Other Hispanic	5.75 (3.77,7.74)	4.77 (2.59,6.96)		3.83 (2.38,5.28)	4.77 (2.59,6.96)	
Others	9.05 (6.97,11.13)	9.12 (6.50,11.75)		8.87 (6.85,10.89)	9.12 (6.50,11.75)	
Marital status			**< 0.0001**			0.85
Married/Living with a partner	63.33 (59.63,67.04)	74.77 (69.79,79.76)		74.38 (67.61,81.15)	74.77 (69.79,79.76)	
Never married	23.87 (20.62,27.12)	12.78 (9.22,16.34)		12.50 (8.43,16.57)	12.78 (9.22,16.34)	
Widowed/Divorced/Separated	12.80 (10.63,14.97)	12.45 (8.65,16.24)		13.12 (8.22,18.02)	12.45 (8.65,16.24)	
Poverty ratio			0.77			0.88
≤1.3	20.16 (16.35,23.96)	18.53 (15.36,21.71)		18.69 (14.24,23.14)	18.53 (15.36,21.71)	
1.4–3.5	35.33 (32.12,38.54)	35.55 (29.83,41.26)		35.30 (29.73,40.87)	35.55 (29.83,41.26)	
>3.5	44.51 (39.74,49.28)	45.92 (39.51,52.33)		46.01 (39.34,52.68)	45.92 (39.51,52.33)	
Education levels			0.12			0.9
High school or less	35.25 (30.82,39.69)	34.52 (28.48,40.56)		36.49 (31.75,41.23)	34.52 (28.48,40.56)	
Some college	28.96 (25.90,32.01)	35.10 (29.18,41.03)		31.58 (26.37,36.79)	35.10 (29.18,41.03)	
College graduate or higher	35.79 (31.45,40.13)	30.38 (24.24,36.52)		31.93 (25.82,38.04)	30.38 (24.24,36.52)	
CVD history			**0.004**			0.77
No	91.66 (90.11,93.21)	86.98 (83.44,90.53)		85.71 (82.55,88.88)	86.98 (83.44,90.53)	
Yes	8.34 (6.79, 9.89)	13.02 (9.47,16.56)		14.29 (11.12,17.45)	13.02 (9.47,16.56)	
Total testosterone, ng/dL	482.05 (472.72,491.37)	232.93 (226.54,239.32)	**< 0.0001**	472.71 (456.62,488.80)	232.93 (226.54,239.32)	**< 0.0001**
Free testosterone, ng/dL	8.42 (8.22,8.62)	5.02 (4.82,5.22)	**< 0.0001**	7.74 (7.44,8.04)	5.02 (4.82,5.22)	**< 0.0001**
LE8 score	70.50 (69.45,71.55)	63.20 (61.26,65.15)	**< 0.0001**	69.82 (68.05,71.59)	63.20 (61.26,65.15)	**< 0.0001**
Health behaviors score	68.58 (67.20,69.95)	66.37 (63.83,68.92)	0.07	69.19 (66.54,71.84)	66.37 (63.83,68.92)	0.11
HEI-2015 diet score	39.25 (36.79,41.72)	35.91 (32.86,38.95)	0.07	40.63 (37.27,43.99)	35.91 (32.86,38.95)	0.06
Physical activity score	80.72 (78.43,83.01)	74.46 (70.92,77.99)	**0.002**	79.42 (75.50,83.33)	74.46 (70.92,77.99)	**0.03**
Nicotine exposure score	69.77 (67.40,72.14)	71.73 (67.33,76.13)	0.42	73.12 (69.35,76.89)	71.73 (67.33,76.13)	0.64
Sleep health score	84.57 (82.82,86.32)	83.40 (80.31,86.50)	0.48	83.59 (80.59,86.59)	83.40 (80.31,86.50)	0.94
Health factors score	72.42 (71.09,73.75)	60.03 (58.22,61.84)	**< 0.0001**	70.45 (68.83,72.08)	60.03 (58.22,61.84)	**< 0.0001**
Body mass index score	66.74 (64.78,68.69)	44.93 (40.92,48.94)	**< 0.0001**	65.03 (62.63,67.43)	44.93 (40.92,48.94)	**< 0.0001**
Blood lipids score	66.15 (63.93,68.37)	56.09 (52.93,59.24)	**< 0.0001**	64.46 (61.57,67.34)	56.09 (52.93,59.24)	**< 0.001**
Blood glucose score	86.90 (85.20,88.60)	77.66 (74.92,80.39)	**< 0.0001**	83.46 (80.37,86.55)	77.66 (74.92,80.39)	**0.002**
Blood pressure score	69.91 (68.28,71.54)	61.45 (57.43,65.46)	**< 0.001**	68.87 (66.73,71.01)	61.45 (57.43,65.46)	**0.001**

Data were presented as weighted percentages or means (95% confidence intervals). Bold P values are significant at P < 0.05. MBAD, male biochemical androgen deficiency; PSM, propensity score matching; CVD, cardiovascular disease; LE8, life’s essential 8; HEI, healthy eating index.

### 3.2 Association of LE8 score with MBAD, TT and FT

Univariate and multivariable analysis before PSM showed that, both as a continuous variable and stratification variables, higher LE8 score as well as LE8 health factors score was associated with decreased risk of MBAD (**Table 2**, all OR < 1, all P < 0.001), elevated level of TT (**Supplementary Table S2**, all β > 0, all P < 0.001) and FT (**Supplementary Table S3**, all β > 0, all P < 0.05). After PSM and concerning the influence of psychiatric disorders, LE8 and LE8 health factors maintained robust results as a protective role for MBAD (**Table 2**; **Supplementary Table S4**).

**Table 2:** Association of the Life’s Essential 8 with MBAD.

Characteristics	Before PSM			After PSM		
	Model 1	Model 2	Model 3	Model 1	Model 2	Model 3
	OR (95% CI), *P*	OR (95% CI), *P*	OR (95% CI), *P*	OR (95% CI), *P*	OR (95% CI), *P*	OR (95% CI), *P*
LE8 score						
Low (0–49)	—	—	—	—	—	—
Median (50–79)	0.37 (0.27, 0.51), **<0.001**	0.37 (0.26, 0.53), **<0.001**	0.36 (0.25, 0.54), **<0.001**	0.47 (0.32, 0.68), **<0.001**	0.43 (0.29, 0.63), **<0.001**	0.39 (0.26, 0.58), **<0.001**
High (80–100)	0.14 (0.08, 0.24), **<0.001**	0.14 (0.07, 0.25), **<0.001**	0.13 (0.07, 0.26), **<0.001**	0.18 (0.11, 0.30), **<0.001**	0.15 (0.08, 0.28), **<0.001**	0.13 (0.07, 0.22), **<0.001**
Continuous	0.96 (0.96, 0.97), **<0.001**	0.96 (0.95, 0.97), **<0.001**	0.96 (0.95, 0.97), **<0.001**	0.97 (0.96, 0.97), **<0.001**	0.96 (0.95, 0.97), **<0.001**	0.96 (0.95, 0.97), **<0.001**
Health behaviors score						
Low (0–49)	—	—	—	—	—	—
Median (50–79)	0.98 (0.70, 1.39), >0.9	0.97 (0.68, 1.39), 0.9	0.96 (0.66, 1.40), 0.8	1.02 (0.65, 1.61), >0.9	0.98 (0.62, 1.56), >0.9	0.95 (0.58, 1.57), 0.8
High (80–100)	0.76 (0.50, 1.13), 0.2	0.68 (0.44, 1.06), 0.087	0.7 (0.43, 1.14), 0.14	0.74 (0.44, 1.23), 0.2	0.69 (0.40, 1.20), 0.2	0.67 (0.38, 1.18), 0.15
Continuous	0.99 (0.99, 1.00), 0.06	0.99 (0.99, 1.00), **0.037**	0.99 (0.98, 1.00), 0.077	0.99 (0.98, 1.00), 0.091	0.99 (0.98, 1.00), 0.07	0.99 (0.98, 1.00), **0.043**
Health factors score						
Low (0–49)	—	—	—	—	—	—
Median (50–79)	0.49 (0.36, 0.66), **<0.001**	0.47 (0.33, 0.66), **<0.001**	0.47 (0.33, 0.68), **<0.001**	0.55 (0.36, 0.84), **0.007**	0.5 (0.33, 0.76), **0.003**	0.49 (0.32, 0.75), **0.003**
High (80–100)	0.14 (0.10, 0.21), **<0.001**	0.13 (0.09, 0.21), **<0.001**	0.14 (0.09, 0.22), **<0.001**	0.19 (0.13, 0.27), **<0.001**	0.15 (0.10, 0.24), **<0.001**	0.14 (0.09, 0.21), **<0.001**
Continuous	0.96 (0.96, 0.97), **<0.001**	0.96 (0.95, 0.97), **<0.001**	0.96 (0.96, 0.97), **<0.001**	0.97 (0.96, 0.97), **<0.001**	0.96 (0.96, 0.97), **<0.001**	0.96 (0.96, 0.97), **<0.001**

Model 1: univariable model. Model 2: adjusted for age, ethnicity and time of venipuncture. Model 3: adjusted for age, ethnicity, time of venipuncture, marital status, poverty ratio, education levels and self-reported cardiovascular disease history. Bold P values are significant at P < 0.05. PSM, propensity score matching; OR, odds ratio; CI, confidence interval.

**Figure 2:**
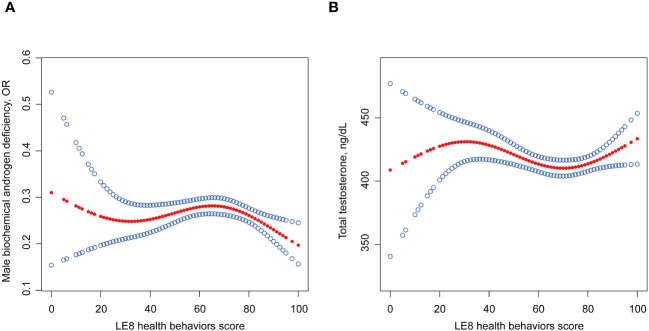
Non-linear relationship of LE8 with **(A)** MBAD and **(B)** TT. Age, ethnicity, time of venipuncture, marital status, poverty ratio, education levels and self-reported cardiovascular disease history were adjusted in this model. LE8, life’s essential 8; MBAD, male biochemical androgen deficiency; TT, total testosterone.

Logistic regression and linear regression showed no association of LE8 health behaviors with MBAD, TT, and FT (**Table 2**; **Supplementary Tables S2**, **S3**). Smooth curve fitting and threshold effect analysis revealed the dose-response association between LE8 health behaviors score with MBAD and TT (**Table 3**, **Figure 2**). When the health behaviors score was below 78.75, it did not indicate a correlation with MBAD and was inversely associated with TT (β -0.55, P = 0.0171). When it exceeded 78.75, it showed a positive correlation with TT (β 1.96, P = 0.0057). However, if it surpassed the threshold of 80, it exhibited a protective effect against both TT and MBAD (OR 0.96, P = 0.0009).

**Table 3:** Threshold effect analysis of LE8 health behaviors score with MBAD, TT and FT.

Outcome:	MBAD	TT	FT
	OR (95% CI), *P*	β (95% CI), *P*	β (95% CI), *P*
Model 1			
Linear effect	1.00 (0.99, 1.00), 0.3518	-0.11 (-0.45, 0.24), 0.5521	-0.00 (-0.01, 0.00), 0.7583
Model 2			
Inflection point (K)	80	78.75	93.75
<K	1.00 (1.00, 1.01), 0.2078	-0.55 (-1.00, -0.10), **0.0171**	-0.00 (-0.01, 0.01), 0.8689
>K	0.96 (0.94, 0.98), **0.0009**	1.96 (0.57, 3.34), **0.0057**	-0.03 (-0.15, 0.10), 0.6563
*P* for log-likelihood ratio	**0.001**	**0.003**	0.665

MBAD, male biochemical androgen deficiency; TT, total testosterone; FT, free testosterone; OR, odds ratio; β, weighted coefficient; Age, ethnicity, time of venipuncture, marital status, poverty ratio, education levels and self-reported cardiovascular disease history were adjusted in this model. Bold P values are significant at P < 0.05.

## 4 Discussions

In this nationally representative cross-sectional study, we found that compared to those without MBAD, participants with MBAD tended to be older and coupled and have a CVD history. Participants without MBAD had higher LE8 score and LE8 health factors score. LE8 score and LE8 health factors score were negatively associated with the prevalence of MBAD. The association remained robust in multiple regression both before and after PSM. Moreover, our results revealed the non-linear association of LE8 health behaviors with MBAD and TT. This is the first study assessing an association between the LE8 and MBAD.

Multiple researches have evaluated the association between testosterone and CVH (23–26). Our discovery aligns with the existing understanding that testosterone is associated with CVH levels. In the present study, the association of LE8 with MBAD and testosterone remained stable after controlling for covariates and sensitivity analyses. This suggested that LE8 is a highly robust independent factor of MBAD and testosterone. The promotion of LE8 contributes to the maintenance of male reproductive function. Besides, many researchers have espoused the view that testosterone at physiological levels confers beneficial effects on the male CVH (1, 11, 27), implying that testosterone may play the role of mediator in LE8 promoting CVH. Another study has demonstrated that maintaining a high level of LE8 would effectively postpone phenotypic aging, with oxidative stress playing a crucial role as a mediator in this biological process (28). This broadened our perception of LE8, suggesting that adherence to the LE8 lifestyle may improve oxidative stress management, thereby reducing inflammation levels and ultimately promoting holistic well-being (29). The decrease in the risk of MBAD is one of the comprehensive optimal outcomes.

The underlying mechanisms of LE8 health factors in endocrine sexual function have been explored. Matteo et al.’s review extensively explored the pathogenesis of Functional Hypogonadotropic Hypogonadism (30). It elucidated that in obese patients, elevated levels of proinflammatory cytokines lead to decreased concentrations of SHBG, ultimately impacting testosterone levels. Moreover, adipose tissue can induce testosterone conversion and affect steroid survival of interstitial cells, resulting in reduced testosterone production. The article also highlights the structural changes in gonads and sexual dysfunction caused by diabetes, with insulin resistance in type 2 diabetes mellitus potentially impairing the secretion of gonadotropin-releasing hormone and decreasing testosterone levels. Another article reported a correlation between sex hormones and arterial hypertension (31). Sex hormones can regulate cardiovascular homeostasis through both slow genomic mechanisms and rapid nongenomic mechanisms, potentially providing cardiovascular protection in premenopausal women (and possibly men).

The present study revealed no significant difference in diet score, smoking score, and sleep score between individuals with MBAD and those without. Besides, there was no linear association between MBAD and LE8 health behaviors score grouped as categorized. To date, several studies on the correlation between metrics of LE8 health behaviors score and testosterone based on linear models have not reached consistent conclusions (32–40). In fact, both excessive and insufficient physical activity are associated with lower testosterone levels (34, 35). There may exist an optimal window of exercise levels where going above or below is associated with adverse outcomes. The evaluation of dietary patterns encompasses various facets, wherein diet exhibits correlations with testosterone levels in certain domains while lacking such associations in others (36–39). A meta-analysis based on 28 studies indicated that testosterone levels are higher in male smokers compared to non-smokers, while no association was found in females. This discovery challenges our preconceived notion of tobacco. The potential mechanism is that tobacco metabolites inhibit testosterone degradation (40). The research findings on the impact of sleep on testosterone levels are also contradictory (32, 33), possibly due to different cutoff values used in sleep assessments. The presence of certain covariates may also contribute to the variability in research results. These studies were unable to draw consistent conclusions about the relationship of physical activity, diet, and smoking with testosterone levels based on the linear model.

However, our study revealed a non-linear association of LE8 health behaviors with MBAD and TT. Only after the health behaviors score reached the threshold of 78.75 and 80 was it protective against TT and MBAD. This may emphasize the lifestyle as a whole, any unhealthy behavior could lead to a bucket effect. Grounded in LE8, physicians can offer the population comprehensive lifestyle guidance in primary healthcare. Testosterone therapy is currently the primary treatment of male MBAD. However, there is currently a lack of comprehensive lifestyle recommendations available for patients with MBAD. LE8 is an assessment tool that can be easily implemented in clinical settings to encourage the adoption of healthy behaviors and optimal health lifestyles. Our study expands upon the existing evidence by demonstrating that ideal CVH metrics not only have a positive impact on CVD but also play a beneficial role in reducing the burden of MBAD. Therefore, adhering to ideal CVH metrics may serve as an effective strategy for preventing and managing MBAD as well as CVD.

This study possesses several strengths. First, the more updated LE8 was utilized to reflect CVH in this study, and the components of the LE8 were analyzed with MBAD, making the findings more comprehensive and targeted. Second, we used a large nationally representative sample of US adults which allows the findings to be generalized to a broader population.

Nonetheless, certain limitations warrant consideration. Firstly, LE8 health behaviors did not demonstrate a correlation with FT in this study. However, AUA guideline recommends that all male patients with TD receive counseling on lifestyle adjustments and consider it as a treatment strategy (3). Therefore, we assume that the lack of correlation between LE8 health behaviors and FT does not hinder clinical practitioners from providing lifestyle recommendations to individuals with MBAD. Secondly, the cross-sectional design of the study precludes drawing causal inferences about the relationship between LE8 and MBAD. Therefore, we hope that readers, when interpreting the study, take into account the potential bidirectional relationship between LE8 and MBAD, especially concerning the metabolic parameters within the Health factors. Then, the potential for recall bias cannot be disregarded due to the utilization of self-report questionnaires to evaluate health behaviors. Another problem arises from the fact that serum testosterone is measured only once, while AUA guidelines recommend 2 levels due to intra-individual and diurnal variations of serum TT. Finally, although we controlled for confounding risk factors of MBAD in regression analyses, unknown or unmeasured confounding variables could still exist.

## 5 Conclusions

In this nationally representative sample of US adults, higher LE8 and its subscales score were independently associated with the lower prevalence of MBAD and higher levels of serum testosterone. A non-linear relationship between LE8 behaviors and MBAD was revealed in the current study. Our findings suggested that LE8 may have the potential to serve as a viable and effective method for promoting male endocrine sexual function. Further research is required to explore the longitudinal and causal relationship between LE8 and the risk of MBAD.

## Data availability statement

The original contributions presented in the study are included in the article/**Supplementary Material.** Further inquiries can be directed to the corresponding author/s.

## Ethics statement

The studies involving humans were approved by National Center for Health Statistics Research Ethics Review Board. The studies were conducted in accordance with the local legislation and institutional requirements. The participants provided their written informed consent to participate in this study.

## Author contributions

WH: Conceptualization, Methodology, Project administration, Writing – original draft, Writing – review & editing. MC: Conceptualization, Methodology, Project administration, Writing – original draft, Writing – review & editing. HZ: Conceptualization, Methodology, Project administration, Writing – original draft, Writing – review & editing. ZZ: Formal analysis, Writing – original draft, Writing – review & editing. CY: Visualization, Writing – original draft, Writing – review & editing. MH: Data curation, Writing – original draft, Writing – review & editing. BS: Funding acquisition, Project administration, Writing – original draft, Writing – review & editing.
