# Supplementary material for: Association between life’s essential 8 and male biochemical androgen deficiency: evidence from NHANES 2013–2016
Source: Front Endocrinol (Lausanne). 2024 Jun 24;15:1369684. doi: 10.3389/fendo.2024.1369684 (PMC11228233; doi:10.3389/fendo.2024.1369684)
Supplement: Supplementary file 1 [file DataSheet_1.docx]

Supplementary Material

**Table S1. Definition and criteria for the American Heart Association’s Life’s Essential 8 score.**

| Domain | CVH Metric | Measurement | Quantification and Scoring of CVH Metric |
| --- | --- | --- | --- |
| Health Behaviors | Diet | Healthy Eating Index-2015 diet score percentile | Quantiles of DASH-style diet adherence  **Scoring (Population):**  Points Quantile  100 ≥95^th^ percentile (top/ideal diet)  80 75^th^ – 94^th^ percentile  50 50^th^ – 74^th^ percentile  25 25^th^ – 49^th^ percentile  0 1^st^ – 24^th^ percentile (bottom/least ideal quartile) |
|  | Physical activity | Self-reported minutes of moderate or vigorous physical activity per week | **Metric:** Minutes of moderate (or greater) intensity activity per week  **Scoring:**  Points Minutes  100 ≥150  90 120 – 149  80 90 – 119  60 60 – 89  40 30 – 59  20 1 – 29  0 0 |
|  | Nicotine exposure | Self-reported use of cigarettes or inhaled nicotine- delivery system | **Metric:** Combustible tobacco use and/or inhaled NDS use; or secondhand smoke exposure  **Scoring:**  Points Status  100 Never smoker  75 Former smoker, quit ≥5 yrs  50 Former smoker, quit 1 - <5 yrs  25 Former smoker, quit <1 year, or currently using inhaled NDS  0 Current smoker  Subtract 20 points (unless score is 0) for living with active indoor smoker in home |
|  | Sleep health | Self-reported average hours of sleep per night | **Metric:** Average hours of sleep per night  **Scoring:**  Points Level  100 7 – <9  90 9 – <10  70 6 – <7  40 5 – <6 or ≥10  20 4 – <5  0 <4 |
| Health Factors | Body mass index | Body weight (kg) divided by height squared (m^2^) | **Metric:** Body mass index (kg/m^2^)  **Scoring:** Points Level 100 <25  70 25.0 – 29.9  30 30.0 – 34.9  15 35.0 – 39.9  0 ≥40.0 |
|  | Blood lipids | Plasma total and HDL-cholesterol with calculation of non-HDL-cholesterol | **Metric:** Non-HDL-cholesterol (mg/dL)  **Scoring:**  Points Level  100 <130  60 130 – 159  40 160 – 189  20 190 – 219  0 ≥220  If drug-treated level, subtract 20 points |
|  | Blood glucose | Fasting blood glucose or casual hemoglobin A1c | **Metric:** Fasting blood glucose (mg/dL) or Hemoglobin A1c (%)  **Scoring:**  Points Level  100 No history of diabetes and FBG <100 (or HbA1c < 5.7)  60 No diabetes and FBG 100 – 125 (or HbA1c 5.7-6.4) (Pre-diabetes)  40 Diabetes with HbA1c <7.0  30 Diabetes with HbA1c 7.0 – 7.9  20 Diabetes with HbA1c 8.0 – 8.9  10 Diabetes with Hb A1c 9.0 – 9.9  0 Diabetes with HbA1c ≥10.0 |
|  | Blood pressure | Appropriately measured systolic and diastolic blood pressure | **Metric:** Systolic and diastolic blood pressure (mm Hg)  **Scoring:**  Points Level  100 <120/<80 (Optimal)  75 120-129/<80 (Elevated)  50 130-139 or 80-89 (Stage I HTN)  25 140-159 or 90-99  0 ≥160 or ≥100  Subtract 20 points if treated level |

**Reference**

1. Lloyd-Jones DM, Allen NB, Anderson CAM, et al. Life's Essential 8: Updating and Enhancing the American Heart Association's Construct of Cardiovascular Health: A Presidential Advisory From the American Heart Association. *Circulation*. Aug 2 2022;146(5):e18-e43.
2. Lloyd-Jones DM, Ning H, Labarthe D, et al. Status of Cardiovascular Health in US Adults and Children Using the American Heart Association's New "Life's Essential 8" Metrics: Prevalence Estimates From the National Health and Nutrition Examination Survey (NHANES), 2013 Through 2018. *Circulation*. Sep 13 2022;146(11):822-835.

**Table S2** Association of the Life’s Essential 8 score with total testosterone.

| **Characteristics** | **Before PSM** | | |  | **After PSM** | | |
| --- | --- | --- | --- | --- | --- | --- | --- |
|  | **Model 1** | **Model 2** | **Model 3** |  | **Model 1** | **Model 2** | **Model 3** |
|  | **β (95% CI), *P*** | **β (95% CI), *P*** | **β (95% CI), *P*** |  | **β (95% CI), *P*** | **β (95% CI), *P*** | **β (95% CI), *P*** |
| LE8 score |  |  |  |  |  |  |  |
| Low (0-49) | — | — | — |  | — | — | — |
| Median (50-79) | 74 (51, 97),  **<0.001** | 67 (42, 93),  **<0.001** | 71 (45, 97),  **<0.001** |  | 51 (27, 75),  **<0.001** | 54 (28, 80),  **<0.001** | 62 (38, 87),  **<0.001** |
| High (80-100) | 135 (102, 168),  **<0.001** | 128 (91, 165),  **<0.001** | 133 (95, 171),  **<0.001** |  | 117 (84, 150),  **<0.001** | 120 (81, 159),  **<0.001** | 133 (100, 167),  **<0.001** |
| Continuous | 2.6 (1.9, 3.2),  **<0.001** | 2.5 (1.8, 3.1),  **<0.001** | 2.7 (2.0, 3.4),  **<0.001** |  | 2.6 (1.9, 3.2),  **<0.001** | 2.7 (2.0, 3.4),  **<0.001** | 3 (2.4, 3.7),  **<0.001** |
| Health behaviors score |  |  |  |  |  |  |  |
| Low (0-49) | — | — | — |  | — | — | — |
| Median (50-79) | -5.5 (-35, 25),  0.7 | -4.9 (-35, 25),  0.7 | 0.67 (-32, 33),  >0.9 |  | -16 (-58, 26),  **0.4** | -14 (-55, 27),  0.5 | -11 (-56, 35),  0.6 |
| High (80-100) | -10 (-39, 18),  0.5 | -2 (-31, 27),  0.9 | 2.8 (-28, 34),  0.9 |  | -3.8 (-48, 41),  0.9 | 0.73 (-46, 47),  >0.9 | 3.2 (-48, 55),  0.9 |
| Continuous | -0.16 (-0.67, 0.34),  0.5 | -0.06 (-0.56, 0.45),  0.8 | 0.04 (-0.49, 0.57),  0.9 |  | 0.11 (-0.67, 0.88),  0.8 | 0.15 (-0.64, 0.94),  0.7 | 0.24 (-0.64, 1.1),  0.6 |
| Health factors score |  |  |  |  |  |  |  |
| Low (0-49) | — | — | — |  | — | — | — |
| Median (50-79) | 55 (34, 75),  **<0.001** | 53 (31, 76),  **<0.001** | 52 (30, 75),  **<0.001** |  | 47 (23, 71),  **<0.001** | 51 (28, 74),  **<0.001** | 53 (29, 77),  **<0.001** |
| High (80-100) | 150 (126, 173),  **<0.001** | 143 (116, 171),  **<0.001** | 139 (113, 165),  **<0.001** |  | 147 (123, 172),  **<0.001** | 153 (128, 179),  **<0.001** | 157 (134, 180),  **<0.001** |
| Continuous | 3.1 (2.7, 3.6),  **<0.001** | 3.1 (2.5, 3.6),  **<0.001** | 3 (2.5, 3.5),  **<0.001** |  | 2.9 (2.5, 3.4),  **<0.001** | 3.1 (2.6, 3.6),  **<0.001** | 3.1 (2.7, 3.5),  **<0.001** |

Model 1: univariable model. Model 2: adjusted for age, ethnicity and time of venipuncture. Model 3: adjusted for age, ethnicity, time of venipuncture, marital status, poverty ratio, education levels and self-reported cardiovascular disease history. Bold P values are significant at P < 0.05. PSM, propensity score matching; β, weighted coefficient; CI, confidence interval.

**Table S3** Association of the Life’s Essential 8 score with free testosterone.

| **Characteristics** | **Before PSM** | | |  | **After PSM** | | |
| --- | --- | --- | --- | --- | --- | --- | --- |
|  | **Model 1** | **Model 2** | **Model 3** |  | **Model 1** | **Model 2** | **Model 3** |
|  | **β (95% CI), *P*** | **β (95% CI), *P*** | **β (95% CI), *P*** |  | **β (95% CI), *P*** | **β (95% CI), *P*** | **β (95% CI), *P*** |
| LE8 score |  |  |  |  |  |  |  |
| Low (0-49) | — | — | — |  | — | — | — |
| Median (50-79) | 1.2 (0.80, 1.6),  **<0.001** | 0.69 (0.31, 1.1),  **0.001** | 0.64 (0.27, 1.0),  **0.003** |  | 0.89 (0.51, 1.3),  **<0.001** | 0.66 (0.28, 1.0),  **0.002** | 0.69 (0.36, 1.0),  **<0.001** |
| High (80-100) | 2 (1.5, 2.5),  **<0.001** | 1 (0.54, 1.5),  **<0.001** | 0.83 (0.32, 1.4),  **0.004** |  | 1.6 (1.1, 2.1),  **<0.001** | 1 (0.50, 1.6),  **<0.001** | 1 (0.47, 1.5),  **0.001** |
| Continuous | 0.04 (0.03, 0.05),  **<0.001** | 0.02 (0.01, 0.03),  **<0.001** | 0.02 (0.01, 0.03),  **0.005** |  | 0.04 (0.03, 0.05),  **<0.001** | 0.03 (0.01, 0.04),  **<0.001** | 0.03 (0.01, 0.04),  **<0.001** |
| Health behaviors score |  |  |  |  |  |  |  |
| Low (0-49) | — | — | — |  | — | — | — |
| Median (50-79) | 0.36 (-0.16, 0.87),  0.2 | 0.32 (-0.09, 0.72),  0.12 | 0.32 (-0.08, 0.71),  0.11 |  | 0.32 (-0.22, 0.86),  0.2 | 0.27 (-0.23, 0.77),  0.3 | 0.25 (-0.28, 0.78),  0.3 |
| High (80-100) | 0.04 (-0.40, 0.48),  0.8 | 0.17 (-0.16, 0.51),  0.3 | 0.08 (-0.28, 0.44),  0.6 |  | 0.51 (-0.02, 1.0),  0.057 | 0.4 (-0.17, 1.0),  0.2 | 0.31 (-0.25, 0.87),  0.3 |
| Continuous | 0 (-0.01, 0.01),  0.6 | 0 (0.00, 0.01),  0.5 | 0 (-0.01, 0.01),  >0.9 |  | 0.01 (0.00, 0.02),  **0.044** | 0.01 (0.00, 0.02),  0.2 | 0.01 (-0.01, 0.02),  0.3 |
| Health factors score |  |  |  |  |  |  |  |
| Low (0-49) | — | — | — |  | — | — | — |
| Median (50-79) | 1.1 (0.78, 1.4),  **<0.001** | 0.65 (0.32, 1.0),  **<0.001** | 0.59 (0.27, 0.92),  **0.001** |  | 0.72 (0.32, 1.1),  **<0.001** | 0.58 (0.18, 1.0),  **0.007** | 0.57 (0.20, 0.94),  **0.005** |
| High (80-100) | 2.4 (2.0, 2.8),  **<0.001** | 1 (0.69, 1.4),  **<0.001** | 0.87 (0.48, 1.3),  **<0.001** |  | 1.9 (1.5, 2.4),  **<0.001** | 1.2 (0.89, 1.6),  **<0.001** | 1.2 (0.86, 1.5),  **<0.001** |
| Continuous | 0.05 (0.04, 0.06),  **<0.001** | 0.02 (0.01, 0.03),  **<0.001** | 0.02 (0.01, 0.03),  **<0.001** |  | 0.04 (0.03, 0.04),  **<0.001** | 0.02 (0.02, 0.03),  **<0.001** | 0.02 (0.02, 0.03),  **<0.001** |

Model 1: univariable model. Model 2: adjusted for age, ethnicity and time of venipuncture. Model 3: adjusted for age, ethnicity, time of venipuncture, marital status, poverty ratio, education levels and self-reported cardiovascular disease history. Bold P values are significant at P < 0.05. PSM, propensity score matching; β, weighted coefficient; CI, confidence interval.

**Table S4** Sensitivity analysis of association of the Life’s Essential 8 score with male biochemical androgen deficiency.

| **Characteristics** | **Model1** | | | **Model2** | | | **Model3** | | |
| --- | --- | --- | --- | --- | --- | --- | --- | --- | --- |
|  | **OR** | **95% CI** | **P** | **OR** | **95% CI** | **P** | **OR** | **95% CI** | **P** |
| LE8 score |  |  |  |  |  |  |  |  |  |
| Low(0-49) | — | — |  | — | — |  | — | — |  |
| Median(50-79) | 0.34 | 0.25, 0.47 | **<0.001** | 0.33 | 0.23, 0.46 | **<0.001** | 0.31 | 0.22, 0.45 | **<0.001** |
| High(80-100) | 0.14 | 0.08, 0.25 | **<0.001** | 0.13 | 0.07, 0.26 | **<0.001** | 0.13 | 0.06, 0.24 | **<0.001** |
| Continuous | 0.96 | 0.96, 0.97 | **<0.001** | 0.96 | 0.96, 0.97 | **<0.001** | 0.96 | 0.96, 0.97 | **<0.001** |
| Health behaviors score |  |  |  |  |  |  |  |  |  |
| Low(0-49) | — | — |  | — | — |  | — | — |  |
| Median(50-79) | 0.99 | 0.68, 1.44 | >0.9 | 0.96 | 0.66, 1.39 | 0.8 | 0.92 | 0.62, 1.38 | 0.7 |
| High(80-100) | 0.75 | 0.50, 1.13 | 0.2 | 0.66 | 0.43, 1.02 | 0.059 | 0.65 | 0.40, 1.07 | 0.082 |
| Continuous | 0.99 | 0.99, 1.00 | 0.087 | 0.99 | 0.99, 1.00 | **0.037** | 0.99 | 0.98, 1.00 | 0.054 |
| Health factors score |  |  |  |  |  |  |  |  |  |
| Low(0-49) | — | — |  | — | — |  | — | — |  |
| Median(50-79) | 0.54 | 0.39, 0.73 | **<0.001** | 0.5 | 0.35, 0.72 | **<0.001** | 0.5 | 0.35, 0.72 | **0.001** |
| High(80-100) | 0.16 | 0.10, 0.25 | **<0.001** | 0.15 | 0.09, 0.24 | **<0.001** | 0.16 | 0.10, 0.25 | **<0.001** |
| Continuous | 0.96 | 0.96, 0.97 | **<0.001** | 0.96 | 0.96, 0.97 | **<0.001** | 0.96 | 0.96, 0.97 | **<0.001** |

Model 1: univariable model. Model 2: adjusted for age, ethnicity and time of venipuncture. Model 3: adjusted for age, ethnicity, time of venipuncture, marital status, poverty ratio, education levels, self-reported cardiovascular disease history and the Patient Health Questionnaire scores. Bold P values are significant at P < 0.05. PSM, propensity score matching; β, weighted coefficient; CI, confidence interval.

**Table S5** Sensitivity analysis of association of the Life’s Essential 8 score with total testosterone.

| **Characteristics** | **Model1** | | | **Model2** | | | **Model3** | | |
| --- | --- | --- | --- | --- | --- | --- | --- | --- | --- |
|  | **Beta** | **95% CI** | **P** | **Beta** | **95% CI** | **P** | **Beta** | **95% CI** | **P** |
| LE8 score |  |  |  |  |  |  |  |  |  |
| Low(0-49) | — | — |  | — | — |  | — | — |  |
| Median(50-79) | 77 | 53, 101 | **<0.001** | 73 | 46, 100 | **<0.001** | 78 | 51, 104 | **<0.001** |
| High(80-100) | 131 | 95, 167 | **<0.001** | 128 | 89, 167 | **<0.001** | 135 | 96, 173 | **<0.001** |
| Continuous | 2.4 | 1.7, 3.1 | **<0.001** | 2.4 | 1.6, 3.1 | **<0.001** | 2.6 | 1.9, 3.4 | **<0.001** |
| Health behaviors score |  |  |  |  |  |  |  |  |  |
| Low(0-49) | — | — |  | — | — |  | — | — |  |
| Median(50-79) | -3.4 | -37, 31 | 0.8 | -1.5 | -35, 32 | >0.9 | 6.2 | -30, 42 | 0.7 |
| High(80-100) | -10 | -43, 23 | 0.5 | 0.36 | -32, 33 | >0.9 | 7.9 | -29, 45 | 0.7 |
| Continuous | -0.25 | -0.80, 0.30 | 0.4 | -0.08 | -0.63, 0.47 | 0.8 | 0.06 | -0.56, 0.67 | 0.8 |
| Health factors score |  |  |  |  |  |  |  |  |  |
| Low(0-49) | — | — |  | — | — |  | — | — |  |
| Median(50-79) | 51 | 29, 73 | **<0.001** | 52 | 27, 78 | **<0.001** | 52 | 25, 78 | **<0.001** |
| High(80-100) | 139 | 114, 164 | **<0.001** | 137 | 107, 167 | **<0.001** | 131 | 104, 159 | **<0.001** |
| Continuous | 3 | 2.5, 3.5 | **<0.001** | 3 | 2.4, 3.5 | **<0.001** | 2.9 | 2.3, 3.4 | **<0.001** |

Model 1: univariable model. Model 2: adjusted for age, ethnicity and time of venipuncture. Model 3: adjusted for age, ethnicity, time of venipuncture, marital status, poverty ratio, education levels, self-reported cardiovascular disease history and the Patient Health Questionnaire scores. Bold P values are significant at P < 0.05. PSM, propensity score matching; β, weighted coefficient; CI, confidence interval.

**Table S6** Sensitivity analysis of association of the Life’s Essential 8 score with free testosterone.

| **Characteristics** | **Model1** | | | **Model2** | | | **Model3** | | |
| --- | --- | --- | --- | --- | --- | --- | --- | --- | --- |
|  | **Beta** | **95% CI** | **P** | **Beta** | **95% CI** | **P** | **Beta** | **95% CI** | **P** |
| LE8 score |  |  |  |  |  |  |  |  |  |
| Low(0-49) | — | — |  | — | — |  | — | — |  |
| Median(50-79) | 1.3 | 0.83, 1.7 | **<0.001** | 0.73 | 0.32, 1.1 | **0.001** | 0.69 | 0.24, 1.1 | **0.006** |
| High(80-100) | 2 | 1.4, 2.5 | **<0.001** | 0.97 | 0.50, 1.4 | **<0.001** | 0.8 | 0.26, 1.3 | **0.007** |
| Continuous | 0.04 | 0.03, 0.05 | **<0.001** | 0.02 | 0.01, 0.03 | **0.002** | 0.01 | 0.00, 0.03 | **0.03** |
| Health behaviors score |  |  |  |  |  |  |  |  |  |
| Low(0-49) | — | — |  | — | — |  | — | — |  |
| Median(50-79) | 0.4 | -0.17, 0.98 | 0.2 | 0.36 | -0.08, 0.80 | 0.1 | 0.38 | -0.09, 0.84 | 0.1 |
| High(80-100) | 0.02 | -0.46, 0.49 | >0.9 | 0.19 | -0.18, 0.55 | 0.3 | 0.12 | -0.31, 0.55 | 0.5 |
| Continuous | 0 | -0.01, 0.01 | 0.8 | 0 | 0.00, 0.01 | 0.7 | 0 | -0.01, 0.01 | 0.8 |
| Health factors score |  |  |  |  |  |  |  |  |  |
| Low(0-49) | — | — |  | — | — |  | — | — |  |
| Median(50-79) | 1 | 0.74, 1.4 | **<0.001** | 0.65 | 0.31, 0.98 | **<0.001** | 0.58 | 0.23, 0.93 | **0.003** |
| High(80-100) | 2.3 | 1.8, 2.7 | **<0.001** | 0.94 | 0.58, 1.3 | **<0.001** | 0.74 | 0.33, 1.2 | **0.002** |
| Continuous | 0.05 | 0.04, 0.06 | **<0.001** | 0.02 | 0.01, 0.03 | **<0.001** | 0.02 | 0.01, 0.03 | **0.003** |

Model 1: univariable model. Model 2: adjusted for age, ethnicity and time of venipuncture. Model 3: adjusted for age, ethnicity, time of venipuncture, marital status, poverty ratio, education levels, self-reported cardiovascular disease history and the Patient Health Questionnaire scores. Bold P values are significant at P < 0.05. PSM, propensity score matching; β, weighted coefficient; CI, confidence interval.

**Table S7** Association of the Life’s Essential 8 score with logarithmic transformed (log2) free testosterone.

| **Characteristics** | **Model1** | | | **Model2** | | | **Model3** | | |
| --- | --- | --- | --- | --- | --- | --- | --- | --- | --- |
|  | **Beta** | **95% CI** | **P** | **Beta** | **95% CI** | **P** | **Beta** | **95% CI** | **P** |
| LE8 score |  |  |  |  |  |  |  |  |  |
| Low(0-49) | — | — |  | — | — |  | — | — |  |
| Median(50-79) | 27 | 15, 39 | **<0.001** | 16 | 5.0, 26 | **0.006** | 14 | 3.4, 25 | **0.013** |
| High(80-100) | 46 | 33, 59 | **<0.001** | 24 | 12, 36 | **<0.001** | 20 | 7.9, 31 | **0.003** |
| Continuous | 0.97 | 0.76, 1.2 | **<0.001** | 0.43 | 0.24, 0.61 | **<0.001** | 0.34 | 0.15, 0.52 | **0.002** |
| Health behaviors score |  |  |  |  |  |  |  |  |  |
| Low(0-49) | — | — |  | — | — |  | — | — |  |
| Median(50-79) | 9.2 | -1.6, 20 | 0.092 | 8 | -0.79, 17 | 0.072 | 6.9 | -2.1, 16 | 0.12 |
| High(80-100) | 7.8 | -1.6, 17 | 0.1 | 10 | 2.5, 18 | **0.012** | 6.5 | -1.5, 14 | 0.11 |
| Continuous | 0.18 | 0.00, 0.36 | 0.053 | 0.18 | 0.04, 0.31 | **0.014** | 0.11 | -0.04, 0.25 | 0.14 |
| Health factors score |  |  |  |  |  |  |  |  |  |
| Low(0-49) | — | — |  | — | — |  | — | — |  |
| Median(50-79) | 20 | 12, 28 | **<0.001** | 10 | 2.4, 18 | **0.013** | 9.3 | 1.5, 17 | **0.023** |
| High(80-100) | 47 | 39, 56 | **<0.001** | 17 | 10, 24 | **<0.001** | 14 | 7.3, 21 | **<0.001** |
| Continuous | 0.94 | 0.77, 1.1 | **<0.001** | 0.32 | 0.17, 0.46 | **<0.001** | 0.27 | 0.13, 0.41 | **0.001** |

Model 1: univariable model. Model 2: adjusted for age, ethnicity and time of venipuncture. Model 3: adjusted for age, ethnicity, time of venipuncture, marital status, poverty ratio, education levels, self-reported cardiovascular disease history and the Patient Health Questionnaire scores. Bold P values are significant at P < 0.05. PSM, propensity score matching; β, weighted coefficient; CI, confidence interval.

**Table S8** Threshold effect analysis of LE8 health behaviors score with MBAD, TT and logarithmic transformed (log2) FT.

| Outcome: | MBAD | TT | Log_2_FT |
| --- | --- | --- | --- |
|  | OR (95%CI), P | β (95%CI), P | β (95%CI), P |
| Model I |  |  |  |
| Linear effect | 1.00 (0.99, 1.00) 0.7278 | -0.20 (-0.58, 0.17) 0.2824 | -0.02 (-0.15, 0.12) 0.8008 |
| Model II |  |  |  |
| Inflection point(K) | 78.75 | 78.75 | 78.75 |
| < K | 1.01 (1.00, 1.01), 0.1393 | -0.59 (-1.07, -0.10), **0.0182** | -0.08 (-0.26, 0.09), 0.3443 |
| > K | 0.97 (0.95, 0.99), **0.0071** | 1.50 (0.06, 2.95), **0.0417** | 0.28 (-0.24, 0.80), 0.2853 |
| P for log-likelihood | **0.006** | **0.016** | 0.239 |

MBAD, male biochemical androgen deficiency; TT, total testosterone; Log_2_FT, logarithmic transformed (log2) free testosterone; OR, odds ratio; β, weighted coefficient; Age, ethnicity, time of venipuncture, marital status, poverty ratio, education levels, self-reported cardiovascular disease history and the Patient Health Questionnaire scores were adjusted in this model. Bold *P* values are significant at *P* < 0.05.


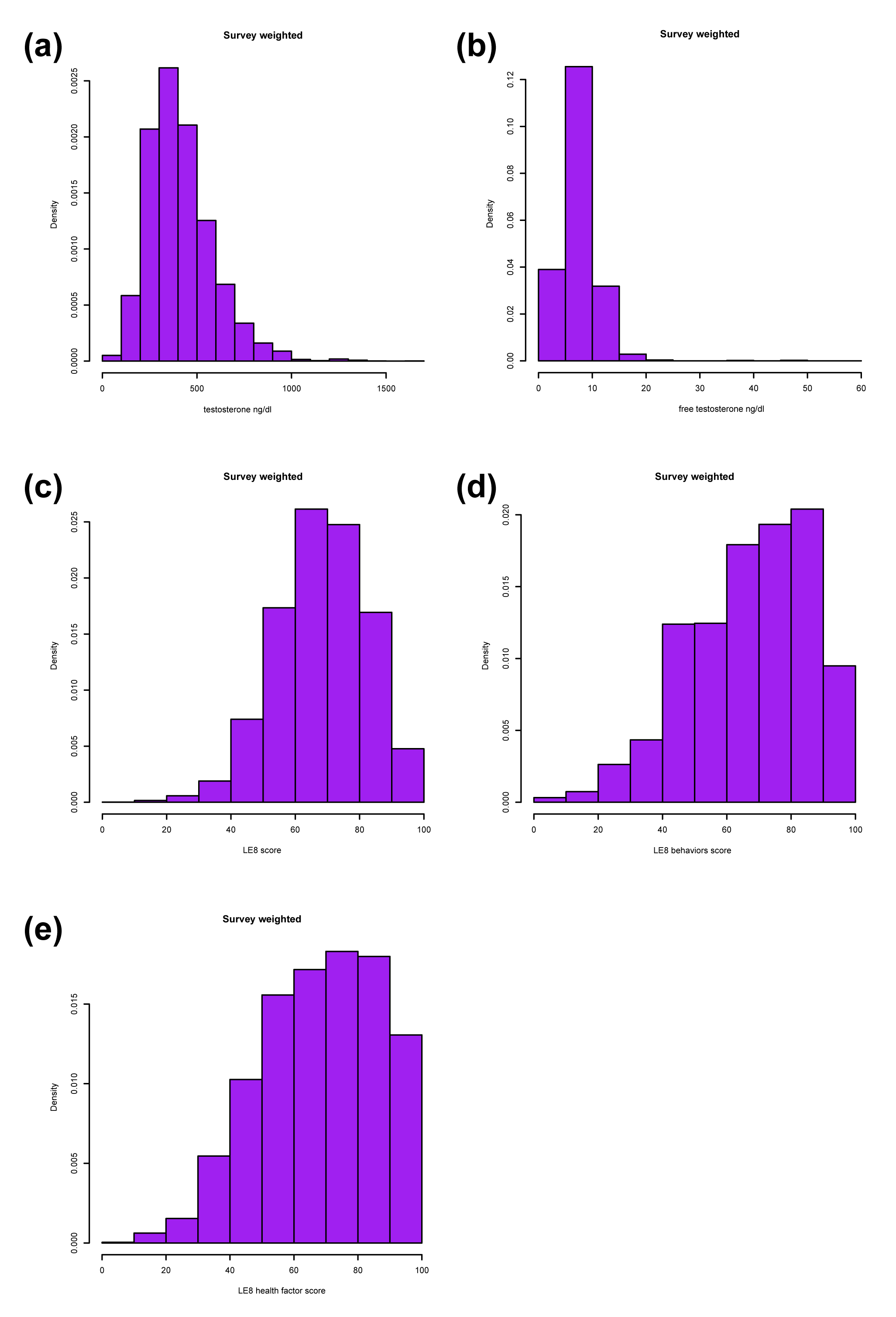


**Figure S1**: The weighted histogram of variables utilized in the study. **(a)**Total testosterone level, **(b)**free testosterone level, **(c)**life’s essential 8 score, **(d)**life’s essential 8 health behaviors score, **(e)**life’s essential 8 health factor score.

**
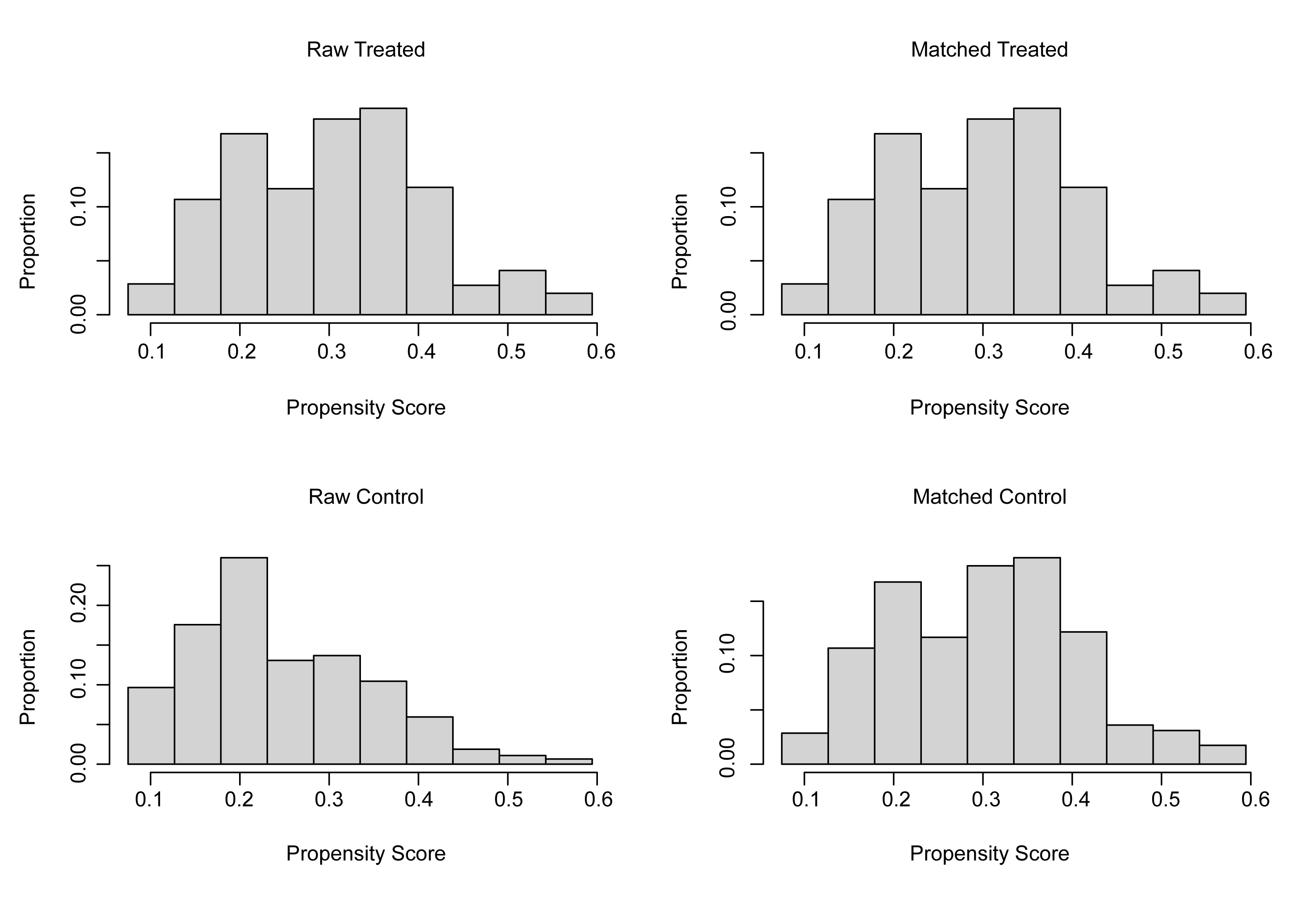
**

**Figure S2**: The histogram of propensity score distribution of the raw data and the matched data. After PSM, the homogeneity was almost achieved in the control and treated groups. PSM, propensity score matching.


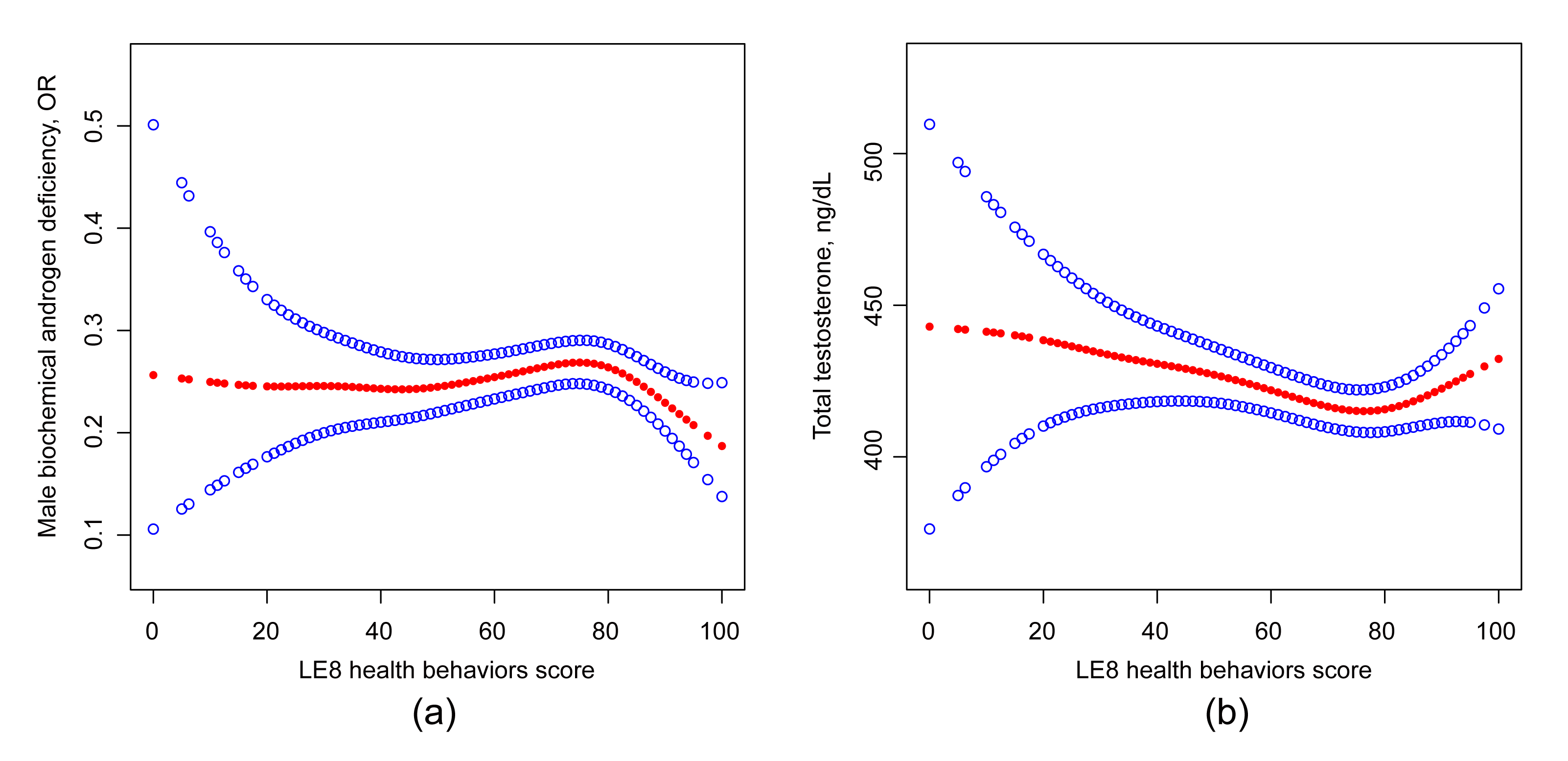


**FIGURE S3**: Non-linear relationship of LE8 health behaviors score with **(a)** male biochemical androgen deficiency and **(b)** total testosterone. Age, ethnicity, time of venipuncture, marital status, poverty ratio, education levels, self-reported cardiovascular disease history and the Patient Health Questionnaire scores were adjusted in this model.
